# Editorial: Monitoring the immune/tumor microenvironment to improve cancer immunotherapy

**DOI:** 10.3389/fimmu.2025.1706172

**Published:** 2025-10-06

**Authors:** Jinhwan Kim, Kelsey P. Kubelick

**Affiliations:** ^1^ Department of Biomedical Engineering, University of California, Davis, Davis, CA, United States; ^2^ Department of Surgery, School of Medicine, University of California, Davis, Sacramento, CA, United States; ^3^ Department of Biomedical Engineering, University of Virginia, Charlottesville, VA, United States

**Keywords:** cancer immunotherapy, tumor microenvironment, biosensor, monitoring tumor, immune cell tracking, monitoring immune responses, theranostics

Although cancer immunotherapies have demonstrated remarkable clinical success across various cancer types, their potential is constrained by heterogeneous patient responses and immune-related toxicities (1). An effective immune response against cancer necessitates the coordinated activity of multiple cellular and molecular mediators within the cancer-immunity cycle, which encompasses antigen release and presentation, immune cell priming, their trafficking and infiltration, and ultimately tumor cell killing (2). Unfortunately, each step can be impacted by the immunosuppressive tumor microenvironment (TME), and failure at any single step can lead to resistance or relapse.

It is important to recognize that the TME is not a static compartment but a dynamic ecosystem where malignant cells, immune effectors, vasculature, and soluble mediators interact in both synergistic and antagonistic ways (3). Traditional methods of studying the immune response within the TME, such as invasive biopsy-based histological analyses or snapshot-like endpoint assays, fail to capture the spatiotemporal dynamics and heterogeneity that shape clinical outcomes (4). Recent advancements in bioimaging, biosensing, and computational modeling now enable us to observe the immune response in real-time, non-invasively, and longitudinally (5, 6). This allows us to map cell-cell interactions *in situ*, monitor therapeutic interventions based on the imaging feedback, and link these observations to patient outcomes. This Research Topic includes 12 contributions (6 original research articles, 3 reviews, 2 mini-reviews, and 1 opinion piece) that collectively illustrate the cutting-edge technologies for visualizing and quantifying the complex spatiotemporal dynamics of the immune-tumor microenvironment. These advanced imaging and diagnostic strategies offer profound insights into mechanisms of resistance, guide the optimization of current cancer immunotherapies, and inspire novel treatment strategies.

A comprehensive review article by Racacho et al. framed the tumor/immune microenvironment (TIME) as a central determinant of cancer progression and therapeutic response, describing how cellular and molecular interactions drive immune activation or suppression and how modern immunotherapies aim to reprogram these processes. Moreover, their study highlighted emerging trends in imaging and artificial intelligence (AI) that enable precise visualization of immune dynamics, setting the stage for research advances in this field. A complementary mini-review article by Purl et al. focused on adoptive cellular therapies for liver metastases, emphasizing how the hepatic niche restricts immune cell trafficking and persistence and how advanced imaging platforms, including PET and MRI, can be leveraged to track and optimize therapeutic cell delivery.

Articles in this Research Topic also emphasized the significance of direct visualization of immune responses. Opinion, review, and research articles by Zhang et al., Zhang et al., and Frecot et al. underscored the need to move beyond static endpoints and adopt spatiotemporal imaging techniques. These techniques, including multiplex tissue imaging, intravital microscopy, and PET tracers targeting CD8 or OX40 to image T cell status, enabled longitudinal and functional monitoring for the identification of earlier and more accurate indicators of therapeutic response.

Advanced imaging strategies have led to the identification of new structural features within the immune-tumor microenvironment. For example, tertiary lymphoid structures (TLS) can serve as organizational centers that underpin therapeutic responses, highlighting their potential role as predictive biomarkers of cancer immunotherapy. In the context of ovarian cancer, a review article by Varghese et al. presented evidence linking TLS, improved survival, and responsiveness to checkpoint blockade therapies. In contrast, a research article by Munoz-Erazo et al. demonstrated how digital pathology methods can be utilized in colorectal cancer to standardize TLS analysis and correlate with clinical outcomes.

Advancements in computational modeling and molecular profiling have further expanded monitoring capabilities. A research article by Liu et al. showcased how a multimodal deep learning framework that integrates pathology, radiology, and clinical data enhanced the prediction of PD-L1 status, immunotherapy response, and survival in esophageal cancer, demonstrating the power of AI-driven data fusion. Complementary bioinformatics and single-cell studies revealed microenvironment-linked biomarkers across tumor types. For instance, research articles by Zheng et al. found RFC4 overexpression in lung adenocarcinoma, Huang et al. identified disulfidptosis/ferroptosis-related gene signatures in endometrial carcinoma, and Miao et al. identified high FGF12 expression in stromal sarcoma. These findings emphasize the importance of interpreting tumor-intrinsic biology through the lens of the immune context. The microenvironment ultimately determines whether molecular changes translate into therapeutic vulnerability.

Finally, Wang et al. provided a mini-review that exemplifies the context-specific nature of immune regulation. Their review focused on IL-37 in gastrointestinal disease, highlighting how this cytokine’s effects vary depending on the tissue and disease stage. This complexity underscores the importance of tailoring immunotherapy strategies to the unique characteristics of local immune environments.

Together, the contributions in this Research Topic highlight how the field of cancer immunotherapy monitoring is evolving through a deeper understanding and real-time assessment of the tumor/immune microenvironment. Articles in this Research Topic demonstrate how non-invasive imaging can capture the dynamics of immune engagement, how to standardize and utilize TLS as actionable biomarkers, how to integrate multimodal approaches and leverage AI to enhance predictive power, how bioinformatics and single-cell approaches can reveal novel immune-linked targets, and how context-specific regulation continues to complicate and enrich our understanding of the tumor/immune microenvironment. Effective cancer immunotherapy demands a united effort to design potent interventions along with tools to monitor, visualize, and guide immune responses as they unfold within the tumor microenvironment.

Finally, we would like to express our gratitude to all of the authors and reviewers for their invaluable contributions to this Research Topic. Their work exemplifies the growing power of interdisciplinary collaboration that integrates immunology, oncology, radiology, engineering, and data science to advance cancer immunotherapy through real-time monitoring of the tumor/immune microenvironment.
